# Experimental-data-driven thermal conductivity prediction and inverse composition design for alloys

**DOI:** 10.1039/d6ra01983h

**Published:** 2026-05-27

**Authors:** Anh D. Phan, Vu Bich Hanh, Ngo T. Que, Nguyen T. T. Duyen, Do T. Nga, Baicheng Mei

**Affiliations:** a Center for Materials Innovation and Technology, VinUniversity Hanoi 100000 Vietnam anh.pd@vinuni.edu.vn adphan35@gmail.com; b College of Engineering and Computer Science, VinUniversity Hanoi 100000 Vietnam; c Faculty of Materials Science and Engineering, Phenikaa University Hanoi 12116 Vietnam; d Institute of Physics, Vietnam Academy of Science and Technology 10 Dao Tan, Giang Vo Hanoi 100000 Vietnam; e School of Materials Science and Engineering, Beijing Institute of Technology Beijing 100081 China

## Abstract

This work develops a data-driven framework for predicting the thermal conductivity of metals and multi-component alloys and for inversely proposing compositions that meet a target conductivity. We collect, to our knowledge, the largest experimental dataset containing 6259 data points spanning 49 elements and temperatures from 0 to 1400 K. Using alloy composition and temperature as inputs, we train and benchmark several regression models and obtain high predictive accuracy reaching *R*^2^ > 0.99 and RMSE of 6–9 W m^−1^ K^−1^. The approach remains quantitatively reliable for challenging cases including dilute-doped Mg alloys and commercial steel over broad temperature ranges. Based on the trained forward model, we propose an inverse-design workflow to efficiently search composition space and suggest candidate alloys expected to achieve a specified thermal-conductivity target at a given temperature. The inverse search can identify composition windows where near-target conductivity is maintained over a finite concentration range to improve the practical ability for experimental validation and scalable process.

## Abstract 2

We collect the largest experimental dataset for alloy thermal conductivity and accurately predict the property from composition and temperature. We can inversely design to find target-matching alloys and composition-tolerant windows for synthesis.

## Graphical abstract

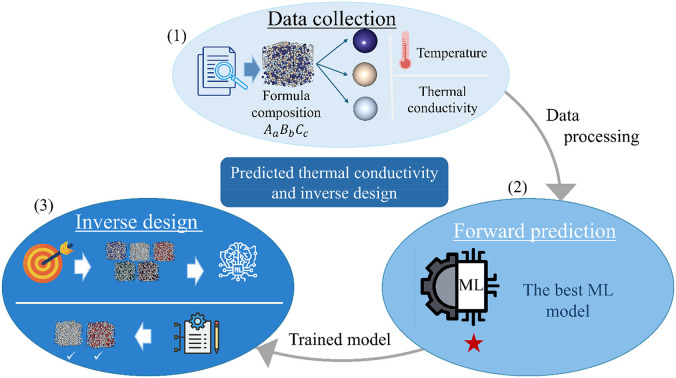

## 1. Introduction

Thermal conductivity (TC) is a fundamental property that describes how efficiently a material conducts heat. Because TC is strongly temperature dependent, it plays a critical role in many technologies including 5 G systems,^1^ electronic devices,^2^ aerospace applications,^3,4^ industrial heat exchangers,^5^ and semiconductor components.^6^ Different applications require different TC ranges. Materials with low TC, such as many polymers and ceramics, are widely used for thermal insulation and thermal barrier coatings, and they are also important in thermoelectric systems.^3,4^ In contrast, metals and alloys typically have high TC and are preferred for heat dissipation and heat transfer in electronics, heat exchangers, and aerospace components.^2,5^ Therefore, the ability to understand and control TC is essential for improving device performance, safety, and long-term reliability.

Experimental methods for measuring thermal conductivity can be typically classified into steady-state and transient categories depending on whether the system reaches thermal equilibrium during the analysis. Steady-state methods, such as the guarded hot plate and heat-flow meter,^7,8^ impose a constant heat flux and measure the temperature gradient. They are widely used for low-conductivity materials and plate-like samples including polymers, thermal insulators, foams, aerogels, and porous ceramics. However, their accuracy is often limited by heat losses, particularly convection and radiation. Thus, these methods are less suitable for high-conductivity materials or thin samples. In contrast, transient methods determine the thermal conductivity from the time-dependent temperature response rather than waiting for steady-state equilibrium. Typical techniques include the hot strip,^9^ hot wire,^10^ transient plane source,^11^ and laser flash^12^ methods. They are generally faster and applicable to a wide range of materials, but their accuracy depends on the assumed heat-transfer model, boundary conditions, and sample homogeneity.

To complement experiments, the thermal conductivity is also investigated using atomistic simulations, most commonly density functional theory (DFT) and molecular dynamics (MD). DFT-based approaches predict the lattice thermal conductivity by combining second- and third-order interatomic force constants with the phonon Boltzmann transport equation,^13–16^ or by using *ab initio* MD with the Green–Kubo formalism.^17^ Although these approaches are physically rigorous, their computational cost limits calculations to relatively small system sizes. This is especially problematic for low-doping materials, where representing dilute impurities without artificial impurity–impurity interactions requires very large supercells. In addition, it is difficult for DFT-based approaches to describe the thermal behaviors in complex, multicomponent, or chemically disordered materials where large-scale structural configurations are essential. Consequently, predicting thermal conductivity in commercial products such as steels and high-entropy alloys, where many alloying elements and microstructural features coexist, remains challenging for simulations. Simulations may require advanced treatments to capture strong anharmonicity at high temperatures. In addition, DFT phonon transport focuses on the lattice term and does not directly account for the electronic thermal conductivity, which is important in metals and many alloys. MD simulations can describe finite-temperature dynamics but its accuracy depends on the quality of the interatomic potential. Moreover, MD simulations do not include quantum statistics of lattice vibrations, which can reduce accuracy at low temperatures.^18–20^ These limitations motivate alternative approaches that can be both accurate and computationally efficient.

Motivated by the cost and practical constraints of experiments and atomistic simulations, machine learning has emerged as an efficient approach for predicting thermal properties. By learning from available datasets, machine-learning models can rapidly estimate the thermal conductivity and allow the high-throughput screening of composition space. However, most ML studies based on experimental alloy data^21,22,25–28^ have been trained on relatively small datasets (typically a few hundred up to ∼1200 samples) and focus on a single alloy family such as Al-based^21^ or Mg-based^22^ systems. Although these models can achieve good accuracy within their training domain, their applications to other alloy chemistries and to different temperature ranges remains uncertain. Other studies rely primarily on MD-based^23^ and larger-scale computational datasets.^24,29^ Such predictions can inherit biases from the underlying computational assumptions and quantitatively differ from experimental data. Several approaches require complex and expert-designed descriptors.^22,25–28^ A recent study^26^ reported promising predictions of temperature-dependent thermal conductivity for additively manufactured metallic alloys. However, its scope is largely limited to a small number of alloy families within a specific processing domain. Moreover, publicly available manufacturer datasheets often provide reference-grade properties because key processing details and product-specific specifications are not fully disclosed. Using such data can introduce variability and bias in model training. Consequently, the applicability of such models and data to broader alloy spaces and wider temperature ranges remains limited.

The above gaps raise several key questions. (1) Can a single ML model trained on experimental data reliably predict thermal conductivity across diverse alloy chemistries covering both low- and high-conductivity regimes over a broad temperature range? (2) How well does such a model generalize to practical materials including dilute-doped systems and commercial materials with complex compositions, where low impurity levels may still produce measurable changes in thermal transport? Can the influence of low impurity concentrations on thermal conductivity be predicted? (3) Can the chemical composition associated with the measurement temperature provide a minimal and transparent input representation with near-experimental accuracy, without relying on expert-engineered descriptors? (4) Can the forward model be exploited as a reliable surrogate for the inverse design, not only suggesting compositions that obtain a target thermal conductivity at a given temperature, but also identifying composition-tolerant “windows” where the target property is maintained under realistic deviations in alloy fractions? Answering these questions calls for a large and diverse dataset with broad temperature coverage, which not only improves predictive reliability but also provides interpretable insights into the key drivers of thermal transport and supports inverse-design approaches that remain practical for synthesis and scale-up.

In this study, we address the above questions by constructing, to our knowledge, the largest experimental dataset currently available for machine-learning prediction of thermal conductivity in metals and alloys. The dataset spans many alloy chemistries at different temperatures and covers a much broader range of thermal conductivity values than prior studies. Using this dataset, we train ML models with chemical composition alone as the input, which reduces model complexity while maintaining high predictive accuracy. Beyond forward prediction, we develop an inverse-design workflow to identify candidate alloy compositions to obtain a target thermal conductivity. Finally, we validate the approach by comparing model predictions with experimental data.

## 2. Method

**Fig. 1:**
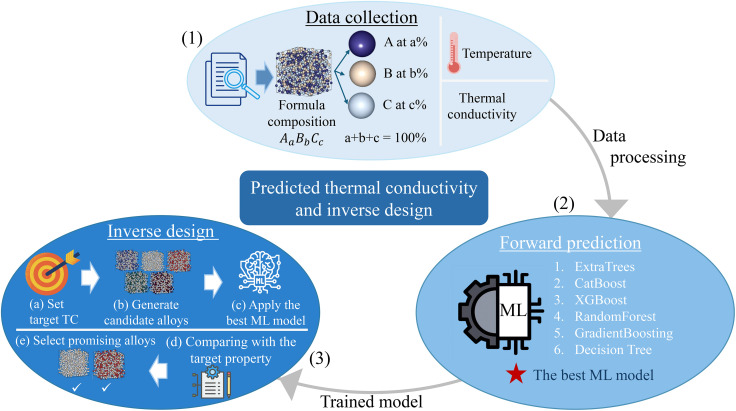
(Color online) The machine learning workflow for forward prediction and inverse design prediction.

Our workflow consists of three stages as shown in Fig. 1. First, we collect experimental thermal-conductivity data for metals and alloys. Second, we train and benchmark six machine-learning algorithms to select the best-performing model for forward prediction. Third, we perform inverse design by sampling candidate alloy compositions within the allowed composition space to determine their thermal conductivity with the selected model and select generated materials having predicted values closest to a specified target.

### 2.1. Data collection and processing

**Fig. 2:**
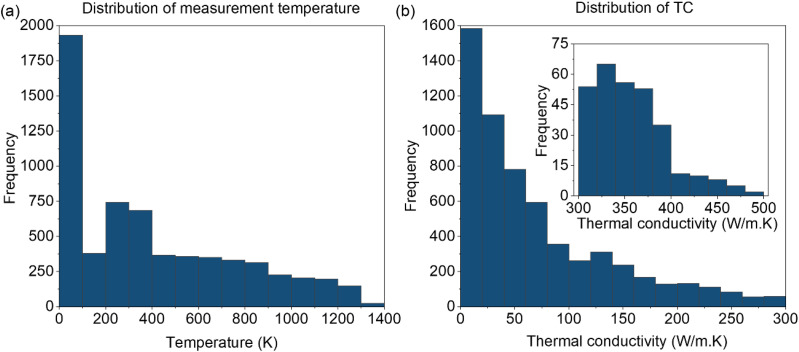
(Color online) Distribution of (a) measurement temperature and (b) thermal conductivity values in the dataset.

We collected an experimental dataset containing 6259 thermal-conductivity measurements for crystalline metals and alloys. The data were extracted from 29 peer-reviewed papers (see SI) and include 49 elements. Because these elements comprise the most commonly used metallic constituents, so the model after training can be applied to a wide range of metallic alloy chemistries of practical interest. The measurement temperatures range from 0 to 1400 K. Fig. 2 presents the distributions of the measurement temperature and thermal conductivity values. The elemental composition distribution of the dataset is provided in the SI. Most measurements are concentrated at low temperatures with the highest counts in the lowest-temperature bins and a long tail extending to 1400 K. Meanwhile, Fig. 2b indicates that the thermal conductivity is also strongly skewed toward low values. The inset of Fig. 2b reveals the sparse high-conductivity tail in the 300–500 W m^−1^ K^−1^ regime.

To improve consistency across sources, we standardized all units and temperature scales, and each alloy composition was converted into a 49-dimensional vector of elemental atomic percentages normalized to sum to 100 $\%$. Entries with missing or ambiguous compositions, inconsistent units, or nonphysical values were removed during manual curation. We note that cross-source differences in processing history, sample form, and measurement procedure are not always reported in sufficient detail to be fully homogenized. Thus, any remaining variability is treated as unavoidable experimental noise and its impact is assessed through validation/test splits and external benchmarks. A representative summary of the dataset is provided in Table 1.

**Table 1:** Examples of processed data structures and corresponding features for five alloys including TC, measurement temperature, and chemical compositions

Alloy	TC (W m^−1^ K^−1^)	Temperature (K)	Al	Ag	Fe	Si	Bi	Sn
Bi_95_Ag_5_	9.61	323	0	5	0	0	95	0
Ag_92.01_Si_7.99_	239.62	732	0	92.01	0	7.99	0	0
Al_94.7_Si_5_Fe_0.3_	165.5	298.15	94.7	0	0.3	5	0	0
Fe_94.91_Al_5.09_	30.8	564.17	5.09	0	94.91	0	0	0
Ag_44.3_Bi_42.9_Sn_12.8_	13.92	373	0	44.3	0	0	42.9	12.8

### 2.2. Machine learning modeling for forward prediction

We begin by reviewing machine-learning models and feature representations used in previous studies^21–29^ as summarized in Table 2. Most previous works have used tree-based ensemble models including Gradient Boosting, Random Forest, XGBoost, CatBoost, and LightGBM and show good predictive accuracy. Other approaches such as SVR/SVM, KNN, linear models, and neural networks including ANN, CNN, LSTM, RNN, and Bayesian neural networks, are less frequently adopted. Based on these works, we focus on tree-based models including Extra Trees, Random Forest, Gradient Boosting, CatBoost, Decision Tree, and XGBoost. The input features consist of the alloy composition (elemental atomic fractions) and the measurement temperature, and the output is the thermal conductivity. For each algorithm, we define a hyperparameter search space and optimize it using GridSearchCV with 5-fold cross-validation.^30^ The hyperparameter grids and the selected optimal values are given in Table S2 of the SI.

**Table 2:** Summary of datasets, data source, range of data, ML/DL models, and test-set performance (R2 and RMSE) for thermal-conductivity prediction in this work and prior studies.^21–29^ Most ML/DL models include Extra Trees (ET), Random Forest (RF), Gradient Boosting (GB), XGBoost (XGB), CatBoost (CB), LightGBM, Support Vector Regression/Machine (SVR/SVM), *k*-Nearest Neighbors (KNN), Linear Regression, Ridge, Lasso, and Stochastic Gradient Descent, Decision Tree (DT), AdaBoost (AB), Stacking Ensemble models, Kernel Ridge Regression (KRR), Artificial Neural Networks (ANN), Recurrent Neural Networks (RNN), Convolutional Neural Networks (CNN), Long Short-Term Memory Networks (LSTM), Gaussian support Vector Regression (rbfs SVR), Feed-Forward Neural Networks, and Bayesian Neural Networks

Size of data	Type of data	Range of data (W m^−1^ K^−1^)	DL/ML model	*R* ^2^	RMSE (W m^−1^ K^−1^)	Reference
271	Experiment	[84, 243]	Gradient boosting	88	12.03	21
			CatBoost	88	12.21	
			XGBoost	91	10.58	
			Stacking ensemble algorithm	83	14.54	
			KNN	50	19.6	
			Linear regression	61	21.98	
			Decision tree	83	14.41	
			AdaBoost algorithm	80	15.73	
			Random forest	84	14.18	
1139	Experiment	[8.1, 167.0]	XGBoost	97.0	—	22
120	MD simulation	∼2, 5	SVR	91.0	1.128	23
79 200	Simulation	∼ [200, 700]	Linear regression	—	101	24
			Ridge regression	—	101	
			LASSO regression	—	101	
			Support vector regression	—	36	
			Feed-forward neural networks	—	7	
			CNN	—	7	
279	Experiment	[0.24, 35]	XGBoost	79.0	1.98	25
			SVM	82.0	1.80	
			KNN	81.0	1.87	
			Kernel ridge	81.0	1.87	
			Gaussian process	81.0	1.86	
294	Experiment	[8.8, 343]	Random forest	92.28	∼2.6	26
			Gradient boosting	90.86	∼2.5	
			XGBoost	96.18	1.63	
			Kernel ridge	69.53	∼4.6	
			Lasso	70.23	∼4.6	
350	Experiment	—	LSTM	88.66	8.36	27
			Linear regression	80.96	26.49	
			Kernel ridge	81.03	26.37	
			Stochastic gradient descent	82.41	17.49	
			Linear SVR	84.79	11.98	
			Sigmoid SVR	94.32	20.61	
			Rbf SVR	75.47	14.62	
			Poly SVR	74.96	12.02	
			Decision tree	53.48	19.37	
			Gradient boosting decision trees	81.58	10.36	
			Random forest	87.67	9.63	
			LightGBM	73.98	12.99	
			ANN	85.93	8.72	
			RNN	87.48	8.37	
			CNN	87.99	8.406	
			Random forest	87.67	9.64	
756	Experiment	[10.9, 83.8]	Bayesian neural network	—	3.9	28
5412	DFT simulation	∼ [0, 115]	Gradient boosting	76.60	7.63	29
6259	Experiment	[0.18, 480]	XGBoost	99.07	9.12	This work

After hyperparameter optimization, the best configuration of each algorithm is evaluated on fixed training, validation, and test sets using a 80 : 20 split. Predictive performance is quantified using the coefficient of determination (*R*^2^) and the root mean square error (RMSE), defined as1
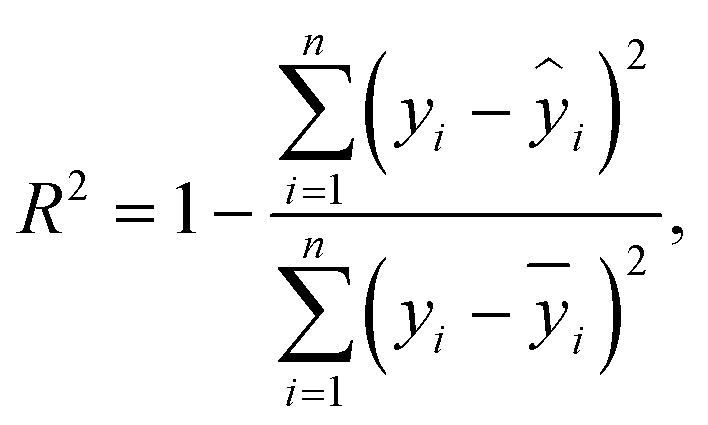
2
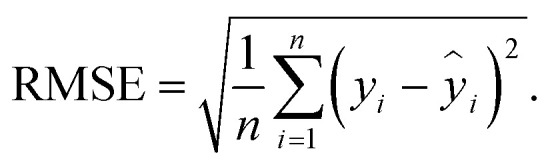
where *y*_*i*_ and *ŷ*_*i*_ are the actual and predicted thermal conductivity for the *i*-th sample, *ȳ* is the mean of the measured values, and *n* is the number of samples. Higher *R*^2^ and lower RMSE indicate better predictive accuracy.

### 2.3. Machine learning modeling for inverse prediction

For inverse design, our goal is to identify alloy compositions whose predicted thermal conductivity is closest to a specified target at a given temperature. Three-million binary alloys are generated by randomly sampling the composition space defined by 49 elements in our dataset to maintain practical feasibility for fabrication. For each alloy, the atomic fractions are constrained to sum to 100%. The thermal conductivity is then predicted by using the trained forward-prediction model for each candidate at the given temperature. We compute the absolute error relative to the target value, rank candidates by this error, and report four candidate alloys with the smallest errors as the most promising designs.

## 3. Results and discussion

### 3.1. Forward prediction of the alloy thermal conductivity

Table 2 benchmarks our results against representative prior studies.^21–29^ Notable differences in dataset size, data source, and conductivity range are shown in Table 2. Experimental datasets are limited to a few hundred to about one thousand samples and focus on a narrow conductivity range. In particular, the prior work^26^ reports 294 measurements involving 20 elements and uses composition and measurement temperature as inputs. Another work^21^ analyzes 271 measurements but includes additional mechanical properties (ultimate tensile strength and yield strength) along with 14 compositional features and temperature. Ref. 28 considers 756 measurements involving 13 elements and temperature. Meanwhile simulation-based studies can provide larger datasets but may differ quantitatively from experiments due to modeling assumptions. In contrast, our dataset contains 6259 experimental data and is in the range of 0.18–480 W m^−1^ K^−1^. This allows us to have good quantitative predictions in both low- and high-conductivity regimes within a unified framework. Across the benchmarks, tree-based ensemble models are the most common predictors among machine/deep learning models. These results suggest that tree-based ensemble models provide accurate prediction of alloy thermal conductivity using only composition and temperature. We also find that this minimal representation remains effective across a much broader experimental alloy space without introducing additional structural or processing descriptors.

**Fig. 3:**
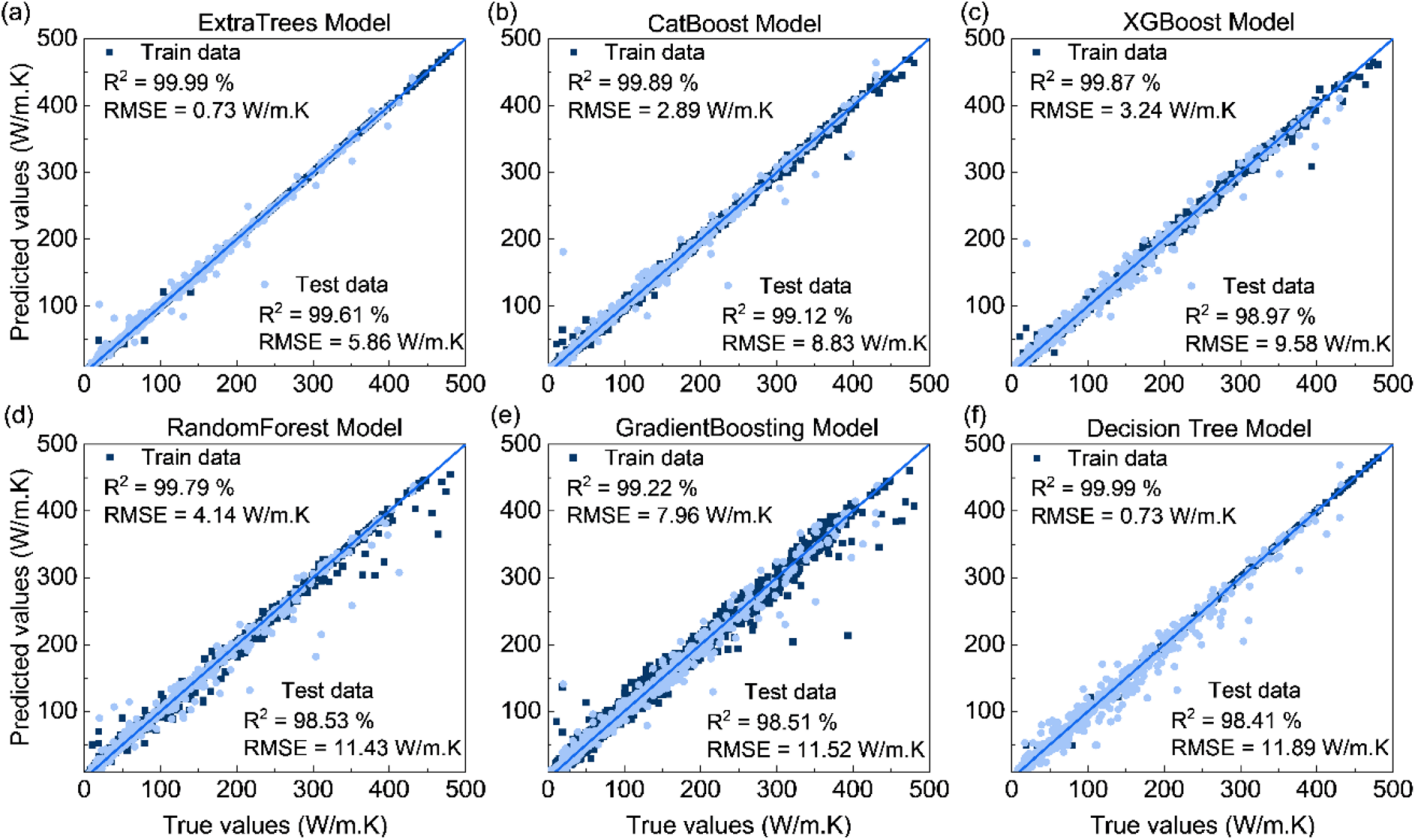
(Color online) Comparison between predicted and actual TC (W m^−1^ K^−1^) values on the training and testing dataset for six machine learning models: (a) Extra Trees, (b) CatBoost, (c) XGBoost, (d) Random Forest, (e) Gradient Boosting, and (f) Decision Tree.

Fig. 3 shows the parity plot between the true and the predicted thermal conductivity on the training and testing dataset. On the training set, the tree-based models reproduce experimental data very closely with most predictions concentrated near the parity line. Extra Trees shows the closest agreement with the training data, and XGBoost, CatBoost, and Random Forest also reproduce the training data well. On the test set, these models retain high accuracy. Extra Trees obtains the best performance with *R*^2^ = 99.61% and RMSE = 5.68 W m^−1^ K^−1^, while CatBoost and XGBoost reach *R*^2^ = 99.12%, RMSE = 8.83 W m^−1^ K^−1^, and *R*^2^ = 98.97%, RMSE = 9.58 W m^−1^ K^−1^, respectively. Gradient Boosting and Random Forest also perform well, whereas Decision Tree exhibits the largest error. These results indicate that alloy composition and measurement temperature contain sufficient information for accurate data-driven prediction of the thermal conductivity. We also evaluated an expanded feature set using Matminer-derived composition descriptors following ref. 29. In this scheme, descriptors are generated directly from the chemical formula by combining elemental properties,^29,31^ including electronegativity, covalent radius, valence electron counts, and periodic-table attributes, into statistics over the constituent elements. This procedure results in 181 input features in total. However, adding Matminer composition descriptors does not improve test-set accuracy compared with the composition–temperature input (see Table S3).

**Fig. 4:**
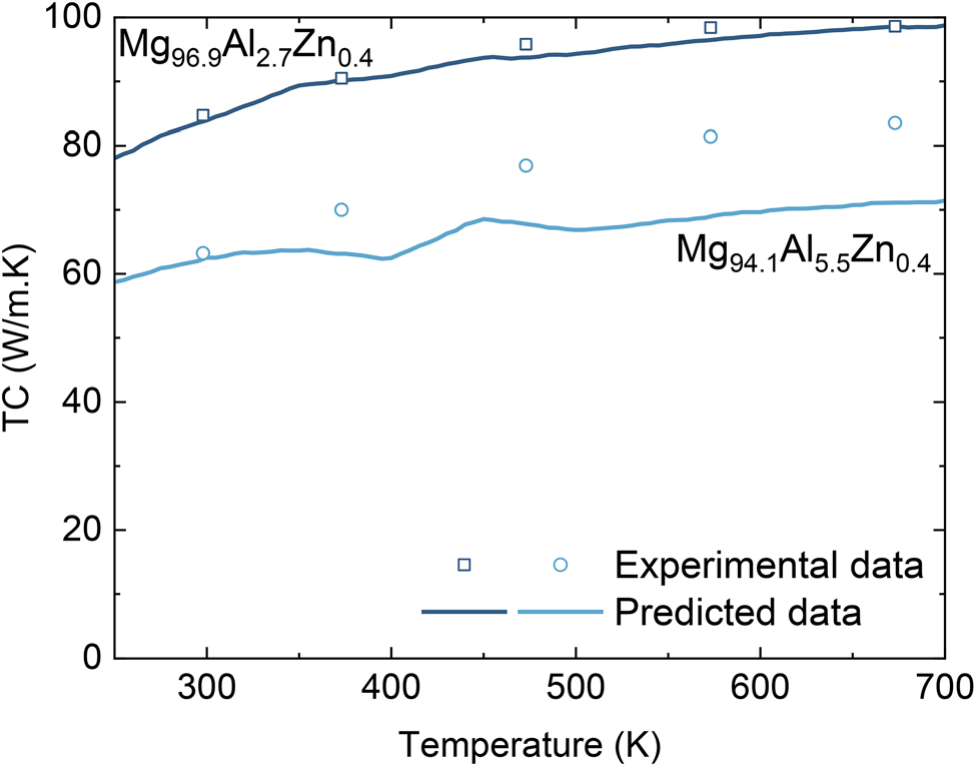
(Color online) Experimental thermal conductivity data (open data points) and our Extra-Trees predictions (solid curves) as a function of temperature for M g96.9Al2.7Zn0.4 and M g94.1Al5.5Zn0.4. Experimental data are taken from ref. 33.

To further validate the Extra Trees model on an external benchmark, we compare its predictions with independent experimental data taken from ref. 32 and 33. As shown in Table 3, the predicted thermal conductivities of Mg_98.82551_Zn_1.17147_Si_0.00106_Ca_0.00130_Fe_0.00067_ agree closely with experimental values^32^ over 348–498 K with deviations of only ∼1–2%. For Mg_99.67488_Al_0_._32440_Si_0.00017_Ca_0.00024_Fe_0.00030_, the model captures the weak temperature dependence of the thermal conductivity in experiment but overestimates the magnitude by about 8–10 W m^−1^ K^−1^ over the same range. We test two additional Mg–Al–Zn alloys, Mg_96.9_Al_2.7_Zn_0.4_ and Mg_94.1_Al_5.5_Zn_0.4_, using different experimental data from ref. 33. The comparisons between our predictions and experimental data are shown in Fig. 4. In all Mg-based cases, the model consistently captures the correct temperature trends and the remaining discrepancies are moderate. These findings suggest that composition and temperature alone can provide reliable screening-level predictions even for dilute alloying additions, while some chemistries may benefit from additional training data or refined descriptors for fully quantitative agreement.

**Table 3:** Experimental thermal conductivity and Extra-Trees predictions at different temperatures for two dilute Mg-based alloys. Experimental data are taken from ref. 32

Temperature (K)	Mg_98.82551_Zn_1.17147_Si_0.00106_Ca_0.00130_Fe_0.00067_		Mg_99.67488_Al_0_._32440_Si_0.00017_Ca_0.00024_Fe_0.00030_	
	Experiment (W m^−1^ K^−1^)	Prediction (W m^−1^ K^−1^)	Experiment (W m^−1^ K^−1^)	Prediction (W m^−1^ K^−1^)
348	123.47	133.63	130.10	130.94
398	122.05	134.51	130.60	131.41
448	125.24	133.35	128.30	129.97
498	125.35	134.21	129.03	130.80

Although we standardize units and compositions across sources, residual variability due to differences in sample form, processing history, and measurement protocols cannot be fully removed because such metadata are not consistently reported. Therefore, part of the prediction error reflects unavoidable experimental noise. The model performance is expected to be strongest in composition–temperature regimes that are well represented in the dataset. It may degrade for sparsely sampled alloy classes such as highly multicomponent high-entropy alloys. Expanding experimental coverage and incorporating relevant metadata when available are expected to further reduce uncertainty and improve quantitative accuracy.

**Fig. 5:**
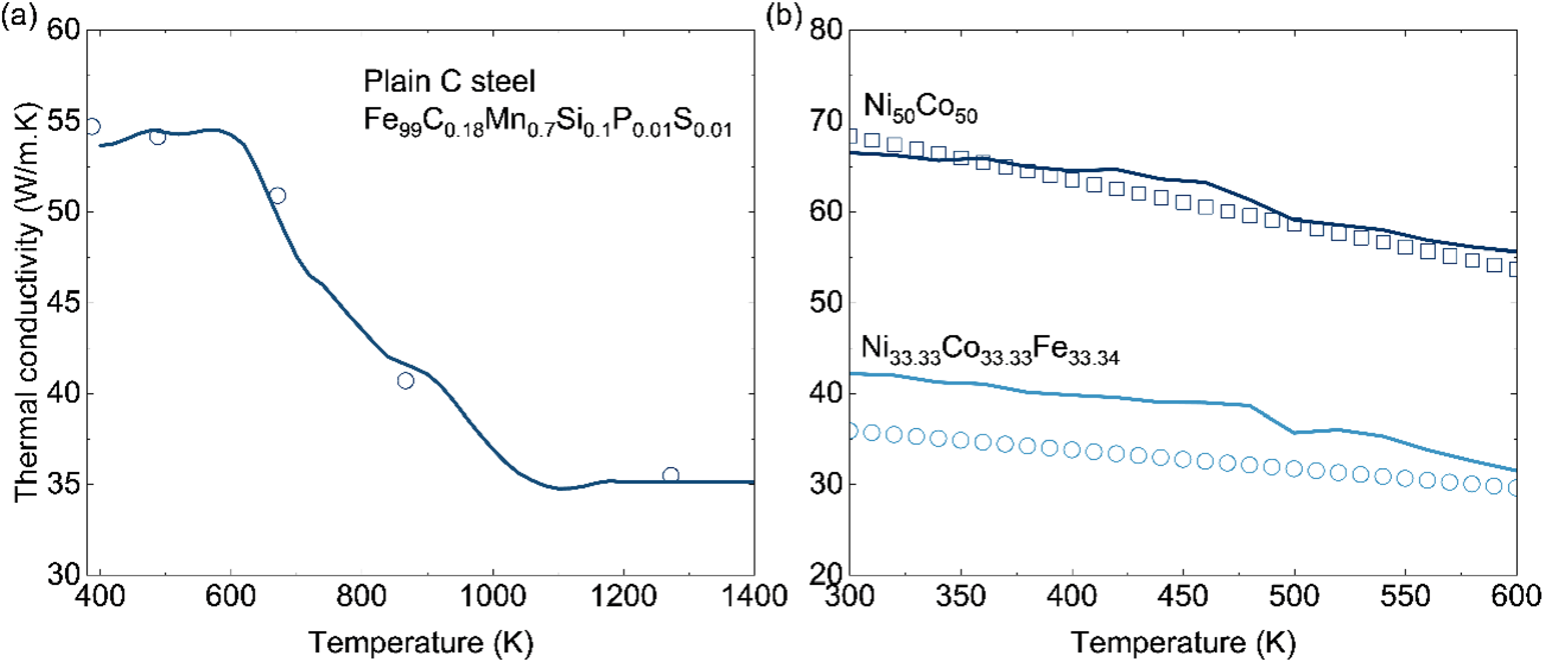
(Color online) Experimental data (symbols) and our predicted thermal conductivity (solid curves) of (a) plain carbon steel (type 316)^35^ and (b) NiCo and NiCoFe alloys.^36^

We now evaluate the Extra-Trees model on a commercial steel with complex and multicomponent chemistry. Fig. 5a compares our predictions with experimental thermal-conductivity data for a plain carbon steel containing minor Mn–Si–P–S additions. Our results show the high conductivity at low temperature, the strong reduction at intermediate temperatures, and the low-conductivity plateau at high temperature. A good quantitative agreement between ML predictions and experiment is observed. The agreement is reliable at low and intermediate temperatures, where the dataset is densest.

In Fig. 5b, we apply the model to mid-entropy alloys Ni_50_Co_50_ and Ni_33.33_Co_33.33_Fe_33.34_. The predicted thermal conductivities agree well with the experimental data and reproduce the monotonic decrease with increasing temperature for both alloys. The model also captures the large separation between the higher-conductivity binary NiCo alloy and the lower-conductivity ternary NiCoFe alloy over a wide temperature range. By contrast, when we tested a high-entropy alloy with more equiatomic components, the model overestimates the thermal conductivity. This is likely due to the limited number of multicomponent materials in the training dataset. Improving prediction accuracy for high-entropy alloys therefore requires more experimental data and remains an important direction for future work.

**Fig. 6:**
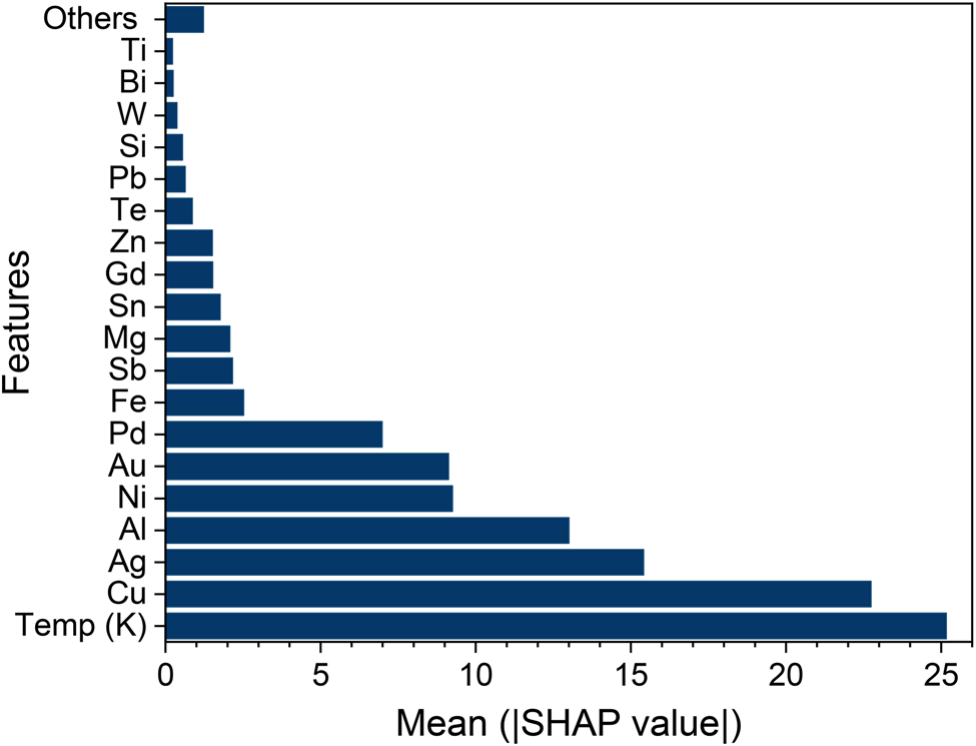
(Color online) Mean SHAP values of all input features.

We assess feature importance using the mean absolute SHAP value, where SHAP (SHapley Additive exPlanations) assigns each input feature a contribution to the model prediction.^34^ As shown in Fig. 6, temperature has the largest impact on the predicted thermal conductivity. This is expected because heat transport in metals and alloys is highly temperature dependent. Increasing temperature enhances phonon scattering and reduces the lattice contribution. While the electronic contribution also changes with temperature because electron–phonon scattering increases and electrical resistivity varies accordingly.

Feature importance analysis reveals that Cu, Ag, Al, and Au have the largest influence on the predicted thermal conductivity. Additionally, Ni, Pd, and Fe are found to contribute significantly to the output. This suggests that fluctuations in concentrations of these specific elements drive the most substantial variations in the thermal conductivity within our dataset. The trend is physically reasonable because Cu, Ag, Al, and Au are inherently high-thermal-conductivity metals. So increasing their content typically elevates the electronic contribution to heat transport and shifts an alloy toward the high-conductivity regime. In contrast, Ni, Pd, and Fe are transition-metal constituents with partially filled d-bands that can strongly affect carrier scattering and electronic structure in alloys. Changes in their concentrations can modify the density of states near the Fermi level and enhance alloy-disorder scattering. These reduce electronic thermal transport and help distinguish lower-conductivity compositions. These mechanisms explain why Cu/Ag/Al/Au and Ni/Pd/Fe emerge as key contributors in the SHAP analysis and why the model can capture transitions between low- and high-conductivity regimes.

### 3.2. Inverse design of the alloy thermal conductivity

In the inverse-design workflow, we generate three million binary alloy candidates by randomly sampling compositions within the 49-element chemical space represented in our dataset. For each candidate, the two elemental atomic fractions are constrained to sum to 100% and the trained Extra Trees model predicts the thermal conductivity at the specified temperature. We quantify each candidate by its absolute error from the target thermal conductivity, sort the candidates by this error, and present four leading design candidates. This procedure allows us to effectively screen a large composition space to identify compositions expected to meet a desired thermal-conductivity requirement. Reliable inverse design therefore depends on the accuracy and generalization of the forward model. As a representative calculation, we target a thermal conductivity of 80 W m^−1^ K^−1^ at 300 K and the inverse-design search identifies four binary candidates: Ag_61.7_Pt_38.3_, Au_58.85_Si_41.15_, Mg_68.87_Bi_31.13_, and W_58.83_Ag_41.17_.

**Fig. 7:**
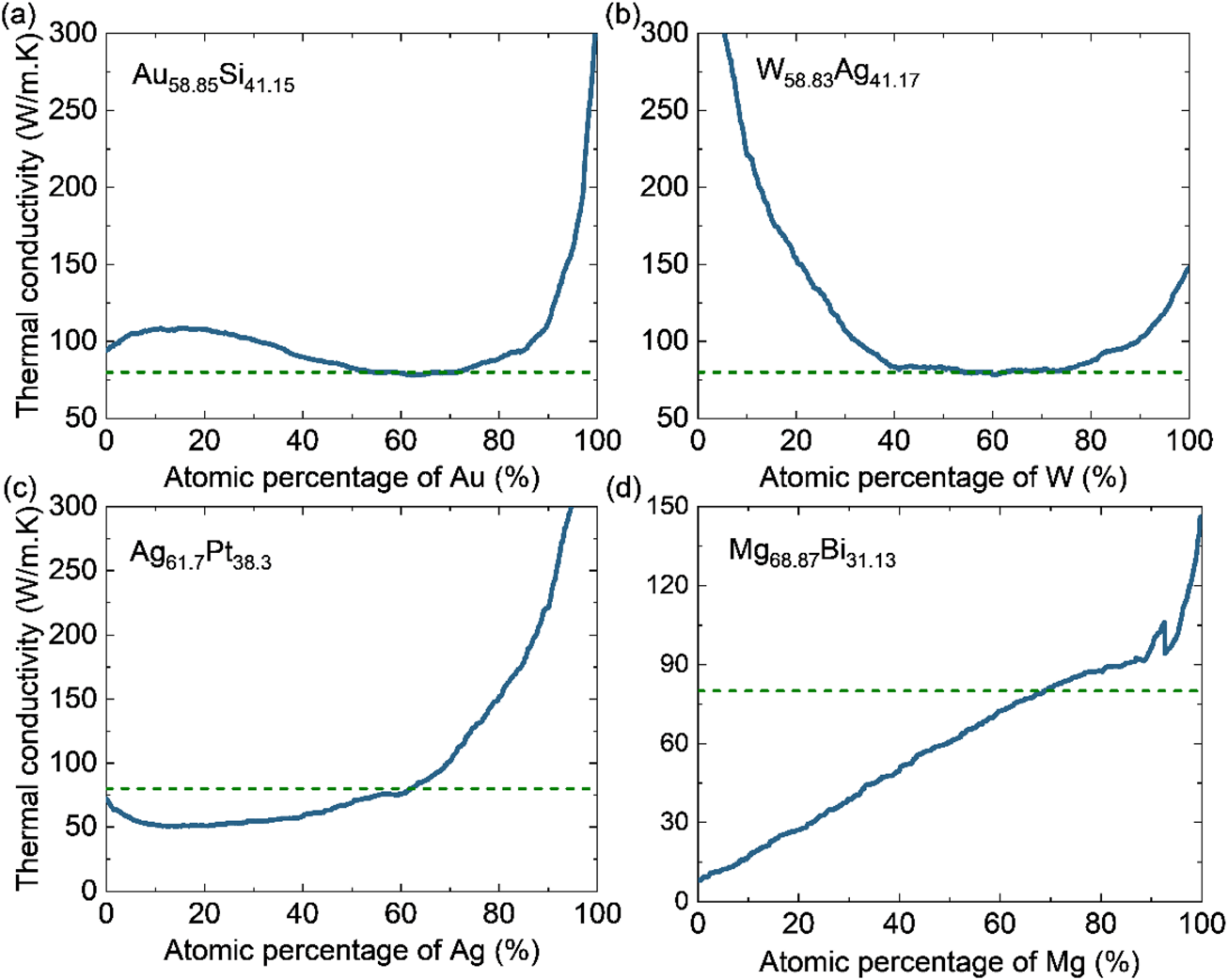
(Color online) The predicted thermal conductivity at 300 K as a function of atomic fraction for the four binary candidates proposed by inverse-design calculations. (a) Au–Si as a function of Au fraction, (b) W–Ag as a function of W fraction, (c) Ag–Pt as a function of Ag fraction, and (d) Mg–Bi as a function of Mg fraction. The green dashed line is the target thermal conductivity used for the inverse design.

A practical question for synthesis is whether an inverse-designed candidate remains close to the target thermal conductivity when its composition deviates slightly from the original recipe. To evaluate this sensitivity, we vary the atomic fraction of one element in each binary candidate and use the trained model to predict the thermal conductivity across the full composition range as shown in Fig. 7. AuSi and WAg alloys exhibit broad plateau-like regions in Fig. 7a and b, where the predicted conductivity is approximately 80 W m^−1^ K^−1^ over a relatively wide interval of Au or W fraction. Such plateaus are interesting for fabrication because modest composition errors are less likely to move the property away from the target. In contrast, AgPt reaches 80 W m^−1^ K^−1^ only within a narrow composition window as shown in Fig. 7c and this reveals that a precious control of the Ag fraction is needed. Meanwhile, MgBi shows an almost monotonic dependence of the thermal conductivity on Mg fraction. A small composition shifts lead to noticeable property changes and, thus, this alloy is a less favorable option for producing an 80 W m^−1^ K^−1^ alloy without high compositional precision. Overall, the inverse-design workflow identifies not only target-matching compositions but also composition-tolerant windows that are better aligned with practical synthesis and scale-up.

## 4. Conclusion

In conclusion, we have successfully built machine learning models to analyze the relationship between chemical composition and the thermal conductivity of metals and alloys. We collect the largest experimental dataset and used chemical composition and measurement temperature as inputs to accurately predict the thermal conductivity without relying on expert-engineered descriptors. Across many models, tree-based ensembles show the best performance on unseen data. In particular, Extra Trees, CatBoost, and XGBoost achieved test-set accuracy around *R*^2^ > 0.99 and RMSE of ∼6–9 W m^−1^ K^−1^. Feature-importance analysis further indicates temperature as the dominant factor and identifies key compositional contributors governing variations in the conductivity within the dataset. External validation on dilute Mg-based alloys shows that the model captures measurable impurity effects and reproduces the temperature dependence of thermal conductivity. The thermal conductivity of Mg_99.67488_Al_0_._32440_Si_0.00017_Ca_0.00024_Fe_0.00030_ is predicted with error of ∼1–2% over 348–498 K. While the thermal conductivity of Mg_99.67488_Al_0_._32440_Si_0.00017_Ca_0.00024_Fe_0.00030_ shows accurate temperature trends but a systematic overestimation in magnitude. It means that certain dilute chemistries may require additional training data or refined descriptors for fully quantitative agreement. Finally, we extended the forward model to inverse design by screening large sets of candidate binary alloys and identifying compositions having a target thermal conductivity at a specified temperature. The inverse search also reveals composition-tolerant windows where predicted conductivity remains near the target over a finite concentration range, which is advantageous for experimental realization and scale-up. This study provides a scalable approach for thermal-transport prediction, composition design, and accelerating materials discovery.

## Conflicts of interest

The authors have no conflicts to disclose.

## Supplementary Material

RA-016-D6RA01983H-s001

## Data Availability

The supporting data has been provided as part of the supplementary information (SI). Supplementary information: Fig. S1 shows the elemental composition distribution of the data. The source code used in this study can be found at Github with https://github.com/NgoQue/ML-Alloys/tree/main. See DOI: https://doi.org/10.1039/d6ra01983h.
