# Supplementary material for: Experimental-data-driven thermal conductivity prediction and inverse composition design for alloys
Source: RSC Adv. 2026 May 27;16(31):28943–51. doi: 10.1039/d6ra01983h (PMC13217163; doi:10.1039/d6ra01983h)
Supplement: RA-016-D6RA01983H-s001 [file RA-016-D6RA01983H-s001.pdf]

Supplementary Information (SI) for RSC Advances.
This journal is © The Royal Society of Chemistry 2026

Supplementary Information

Experimental-Data–Driven Thermal Conductivity Prediction and Inverse Composition Design for Alloys

Anh D. Phan,[1,2,*] Vu Bich Hanh,[3] Ngo T. Que,[1] Nguyen T. T. Duyen,[3] Do T. Nga,[4] and Baicheng Mei[5]

[1]Center for Materials Innovation and Technology, VinUniversity, Hanoi 100000, Vietnam

[2]College of Engineering and Computer Science, VinUniversity, Hanoi 100000, Vietnam

[3]Faculty of Materials Science and Engineering, Phenikaa University, Hanoi 12116, Vietnam

[4]Institute of Physics, Vietnam Academy of Science and Technology, 10 Dao Tan, Giang Vo, Hanoi 100000, Vietnam

[5]School of Materials Science and Engineering, Beijing Institute of Technology, Beijing 100081, China

Alloy	Ref
Pt<br>Pt-Ni	10.1016/S0925-8388(98)01012-1
Al-Si-Fe-Cu-Ni-Zn	10.1007/s10853-015-9115-9
Al-Cu, Au-Ag, Fe-Ni<br>Cu-Zn, Ag-Pd, Ag-Pd<br>Cu-Pd, Al-Mg, Cu-Ni<br>Cu-Pd, Cu-Au	10.1063/1.555583
Co-Ni	10.1016/j.jmst.2020.04.045
Al-Si-Mn-Mg-Fe-Ti-Sr-Ni-Cr-Cu-Zn-Zr	10.3390/ma17125329
Ti-Al-V-Mo-Cr-Zr-Fe-C-O-N	10.1134/S0031918X14050020
Cu-Cr-Zr-Ti-Fe	10.5402/2012/580654
Mg-Zn-Mn	10.1016/j.jallcom.2013.03.184
Mg-Mn-Fe-Si	10.1016/j.jallcom.2015.11.107
Al-Si-Fe-Cu-Ni-Zn	10.1007/s10853-015-9115-0
Fe-Al	10.2355/isijinternational.42.322
Al-Fe, Al-Fe-Ce	10.1016/S1003-6326(22)65908-9
Co-Sb-S-Se	10.1038/srep46630
Al, Cu, Ag, Au, W, Mo, Ni<br>Fe, Pt, Pd, Y, Mg, Ti, Cr, Co, Sn	NIST NSRDS 8 (PDF)
Al-Cu, Al-Si	10.1016/j.acta.2016.01.019
Ag-Bi-Sn	10.1016/j.tca.2022.179344
Al-Fe, Al-Si, Al-Si-Fe	10.1007/s10962-021-00580-0
Ag-Si	10.1016/j.jpcs.2021.109997
Bi-Ag	10.1007/s10973-020-10482-8
Mg-La-Zr	10.1016/j.jallcom.2017.10.013

Pb-Li	10.1016/j.fusengdes.2015.12.029
Na-Sn-Bi-Te	10.1021/jacs.0c05650
W-Re	10.1016/S0167-577X(02)01403-9
Pt-Ni	10.1016/S0925-8388(98)01012-1
Mg-Al	10.1007/s10765-013-1490-3
Mg-Al, Mg-Sn, Mg-Zr<br>Mg-Mn, Mg-Ca	10.1007/s10853-013-8012-3
Al-Gd-Cu, Al-Gd-Ni, Al-Gd-Mn<br>Al-Gd-Co, Al-Gd-Fe, Al-Gd-Ta<br>Al-Gd-Zr, Al-Gd-Mo, Al-Gd-Ti	10.1016/S1003-6326(15)63798-0
Zr-Hf-Ni-Sn-Sb	10.1039/C5TA04184A
Zr-Ni-Sn, Ti-Ni-Sn<br>Zr-Ti-Ni-Sn, Hf-Ti-Ni-Sn	10.1038/s41598-017-14013-8

Table SII: Summary of the optimized hyperparameters for different machine-learning models.

Model	Hyperparameters
Extra Trees	max depth=None; min samples split=2; n estimators=300
CatBoost	depth=8; iterations=800; l2 leaf reg=1; learning rate=0.1
XGBoost	max depth=7; learning rate=0.1; subsample=0.8; colsample bytree=1.0
Random Forest	max depth=None; min samples split=2; n estimators=500
Gradient Boosting	max depth=4; learning rate=0.1; n estimators=500
Decision Tree	max depth=None; min samples split=2

Table SIII: Performance comparison of machine-learning models using the extended 181-feature descriptor set.

Model	R^2 (%) train	RMSE train	R^2 (%) train	RMSE train
CatBoost	99.96	1.68	98.89	9.91
Extra Trees	99.99	0.73	98.77	10.50
XGBoost	99.96	1.76	98.01	13.33
Random Forest	99.81	3.93	97.75	14.18
Gradient Boosting	99.64	5.41	97.71	14.31
Decision Tree	99.99	0.73	95.74	19.52

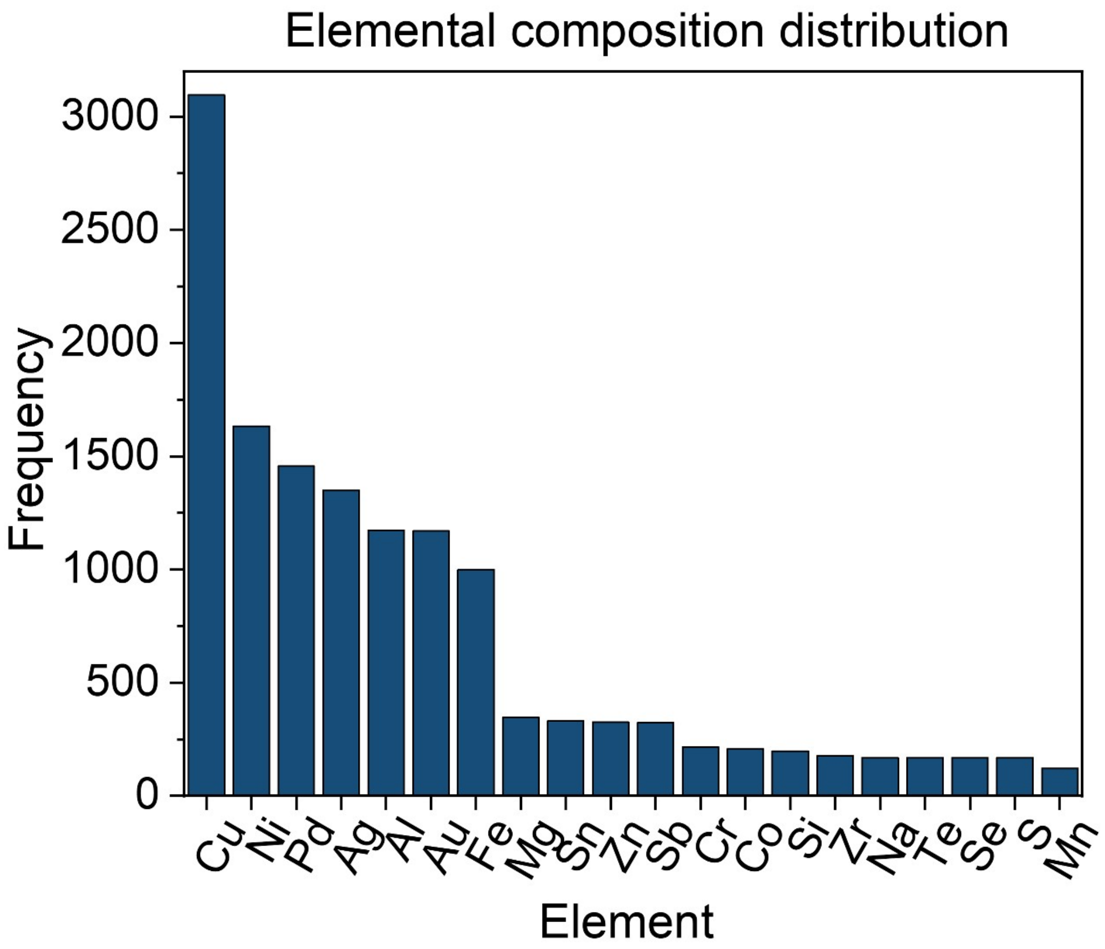

Figure S1: (Color online) Top 20 most common elements in the dataset and their occurrence frequencies
